# Influence of orthodontic treatment with premolar extraction on the spatial position of maxillary third molars in adult patients: a retrospective cohort cone-bean computed tomography study

**DOI:** 10.1186/s12903-020-01314-0

**Published:** 2020-11-11

**Authors:** Fangwei Pan, Zhentao Yang, Jian Wang, Ruilie Cai, Jialing Liu, Chenghao Zhang, Wen Liao

**Affiliations:** 1 State Key Laboratory of Oral Diseases and National Clinical Research Center for Oral Diseases, West China School of Stomatology, Sichuan University, Chengdu, Sichuan China; 2 State Key Laboratory of Oral Diseases and National Clinical Research Center for Oral Diseases, Department of Prosthodontics, West China Hospital of Stomatology, Sichuan University, Chengdu, Sichuan China; 3 Department of Epidemiology and Biostatistics, Arnold School of Public Health, University of South Carolina, Columbia, SC USA; 4 State Key Laboratory of Oral Diseases and National Clinical Research Center for Oral Diseases, Department of Orthodontics, West China Hospital of Stomatology, Sichuan University, Chengdu, Sichuan China

**Keywords:** Cone-bean computed tomography, Molar, third, Maxilla, Bicuspid, Tooth extraction, Spatial position, Adult

## Abstract

**Background:**

Based on low-dose radiation Cone-bean computed tomography (CBCT) images, This study aims to establish a space coordinate system, which offers more precise and comparable evaluation on changes of maxillary third molars influenced by orthodontic treatment with premolar extraction in adults. The system suggests promising application prospect in future studies related to CBCT superimposition and evaluation for its feasibility and efficiency.

**Methods:**

Forty-nine maxillary third molars from 27 patients (mean age, 20.78 years) were included. CBCT images were obtained before and after orthodontic treatment with premolars extracted (mean treatment duration, 31.47 months). The changes in the position, angulation, and rotation of the third molars were evaluated with a space coordinate system using four landmarks: anterior nasal spine (ANS), posterior nasal spine (PNS), left and right orbitales.

**Results:**

After orthodontic treatment, the third molars moved forward (adjusted mean, 1.44 mm) (*p* < 0.001) and downward (adjusted mean, 2.87 mm) (*p* < 0.001) accompanied by outward rotation of the crowns (adjusted mean, 5.38°) (*p* = 0.001), while changes in angulation were insignificant.

**Conclusions:**

This was the first study to systematically investigate the spatial position change of maxillary third molars in adult patients who received orthodontic treatment with premolar extraction. During the process, maxillary third molars moved downward and forward accompanied by outward rotation of the crowns. Orthodontists should take tooth movement potential into consideration when making extraction plans.

## Background

Extraction of premolars, as a routine orthodontic treatment for reducing protrusion or crowding of dental arch, has been widely used in the correction of severe tooth-arch discrepancy and, in certain cases, sagittal dysmorphosis. 8.9–13.4% of orthodontic treatments included four first premolar extractions, with an overall extraction frequency fluctuating around 25% (third molars excluded) [1]. In East Asia, the extraction rate is even higher, because of severer alveolar bone-dental arch discrepancy of Mongoloids [2].

Third molars are reported with the highest frequency of impaction [3] and impacted third molars are closely related to complications [4], among which possible late incisor crowding and post-orthodontic relapse are main concerns of orthodontists [5]. Therefore, many asymptomatic third molars were extracted for prophylactic purposes on dentists’ personal values and empirical basis [6]. However, considering the possibility of iatrogenic injury [7], economic burden [8], potential for auto-transplantation [9] and psychological anticipation of patients, other orthodontists recommend more conservative approaches. Thus, the management of third molars, especially their extraction, has long been a matter of debate [10].

In theory, the extraction of premolars could relieve posterior space discrepancies. With mesial movement of the buccal segment, the position and angulation of third molars would change, which has the potential to ameliorate potential impacted situations and minimize possible risks. A systematic review published in 2017 [11] demonstrated favorable changes in the eruption rate, retromolar space, and angulation of third molars, especially in maxillary. Regrettably, due to various reference lines and inconsistent measurements, the level of evidence was relatively low.

Measuring three-dimensional objects using two-dimensional images routinely leads to inevitable errors [12, 13]. Previous similar studies have all been based on two-dimensional images (lateral cephalogram or panoramic radiograph). With widely application in clinic and research, CBCT is promised to establish a uniform and all-sided measurement system thus improving the reliability and comparability of results. Further, adult patients are preferred to distinguish the effect of therapeutic orthodontic intervention from that of natural growth. Last but not least, adequate description of vital characteristics of selected cases, such as anchorage, is crucial. Thus, a robust meta-analysis to guide clinical decisions is obtainable.

This study established a handy space coordinate system which allows for further superimposition based on CBCT images to precisely evaluate the changes of maxillary third molars influenced by orthodontic treatment with premolar extraction. Position, angulation, and rotation of the maxillary third molars pre- and post-treatment were measured and compared. Further, we expect promising application prospect of the system in future studies related to CBCT superimposition and evaluation for its feasibility and efficiency.

## Methods

This is a retrospective cohort study and patients who received orthodontic treatment in the Department of Orthodontics, West China Hospital of Stomatology, Sichuan University (Chengdu, China) from March 2014 to January 2020 were filtered manually in a medical record database of the hospital.

The sample size was calculated according to Lee [14], and by setting the level at 0.05 and power at 0.9, at least 24 samples were needed. Designed with before-after contrast, this study needed at least 12 teeth.

The inclusion criteria were: (1) patients with CBCT images taken before orthodontic treatment (T1) and after the treatment (T2) were available, (2) patients who were 18 to 30 years of age at T1, (3) patients with one or two maxillary premolars extracted during orthodontic treatment, and (4) patients with third molar images in relevant tooth extraction sites existed in both pretreatment and posttreatment CBCT images. The exclusion criteria were: (1) patients with craniofacial syndrome and systemic disease, (2) patients with CBCT images taken 2 weeks before the treatment began or 2 weeks after the treatment finished, and (3) patients with insufficient CBCT image quality, that is, any critical landmark was missing.

Orthodontic diagnoses and treatment characteristics of selected patients were recorded according to the medical records combined with pretreatment CBCT images.

All the CBCT images were taken within 2 weeks before and after the orthodontic treatments. The standards of the end of orthodontic treatment were fully closed space and functional occlusal relationship. All the CBCT images were taken using the same CBCT machine (3D Accuitomo, Morita Group, Japan), which was set according to the manufacturers’ recommendations (10*10 cm FOV, 85 kV, 4 mA, and 360° rotation). The voxel size is 125 μm. During image acquisition, patients were seated statically with the Frankfort plane parallel to the ground. The CBCT data were exported in DICOM multifile format and imported into *Invivo* software (Version5.3.4; Anatomage, Inc., San Jose, CA, USA) with the plugin “3D analysis” for 3D volume rendering and later evaluation.

**Fig. 1:**
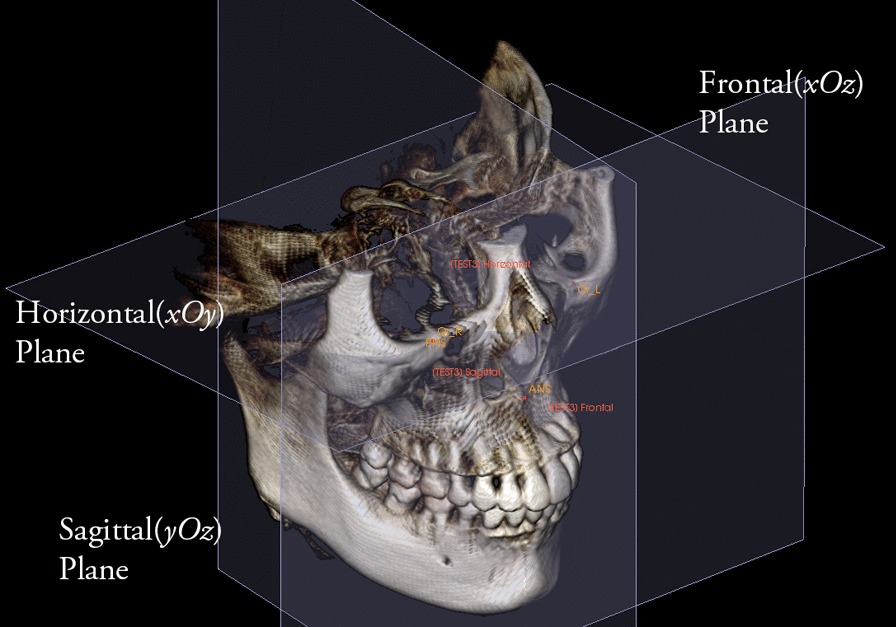
Maxillary coordinate system shown with 3 planes: horizontal(*xOy*), frontal(*xOz*) and sagittal(*yOz*) planes. 4 landmarks were used for setting up the system: ANS, anterior nasal spine; PNS, posterior nasal spine; Or_L, left orbitale; Or_R, right orbitale

**Fig. 2:**
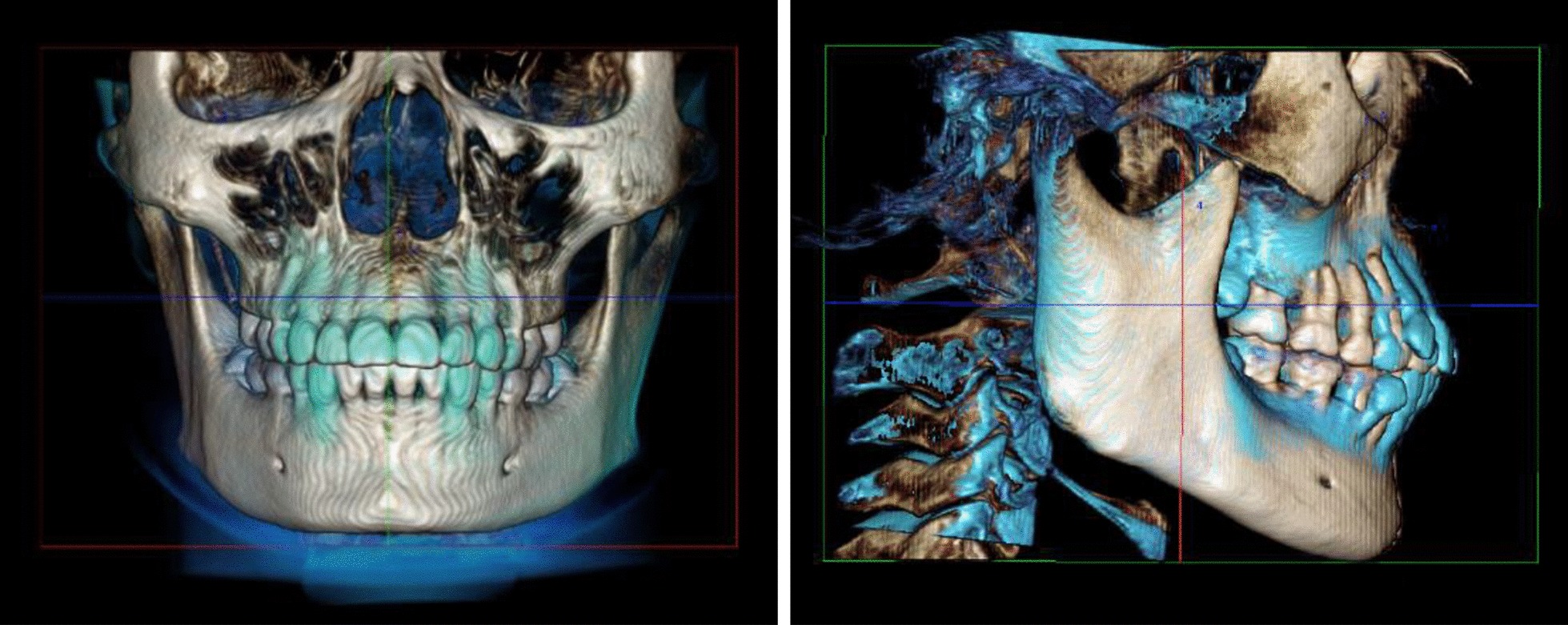
Landmark CBCT superimposition profiles. The same 4 landmark were included: ANS, PNS, bilateral orbitales

A space coordinate system was used with four landmarks as follows (Fig. 1). The horizontal plane (xOy) was defined as the plane passing through bilateral orbitales, while parallel to ANS-PNS. The sagittal plane (yOz) was defined as the plane passing through ANS and PNS while perpendicular to the horizontal plane. The frontal plane (xOz) was defined as the plane perpendicular to both horizontal plane and sagittal plane while passing through ANS. Landmark superimposition with the same four landmarks was performed to overlap three-dimensional reconstructed pre- and post- therapy images to evaluate the stability of the space coordinate system (Fig. 2).

**Fig. 3:**
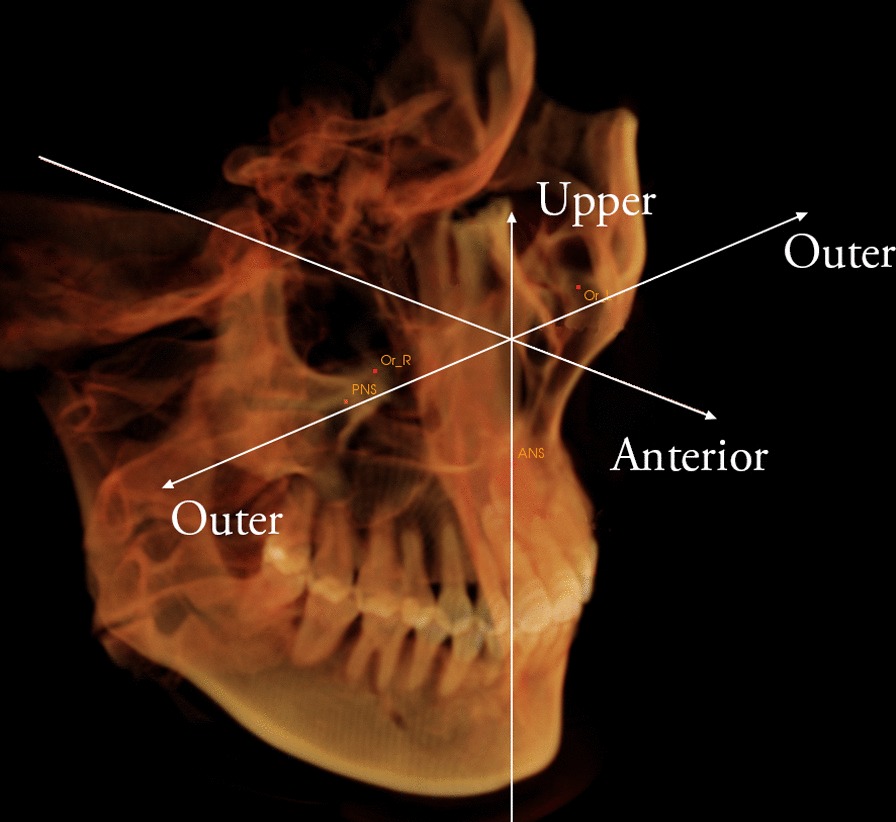
Upper, anterior, outer directions were defined as positive directions

Six other landmarks were located to define the spatial position of the third molar: mesiobuccal and distobuccal cusps of the third molar, root furcation and central pit of the third molar, root furcation and central pit of the second molar. Software calculated the linear and angular dimensions between certain landmarks as follows. The forward, outward, and upward position were defined as positive directions (Fig. 3).

**Fig. 4:**
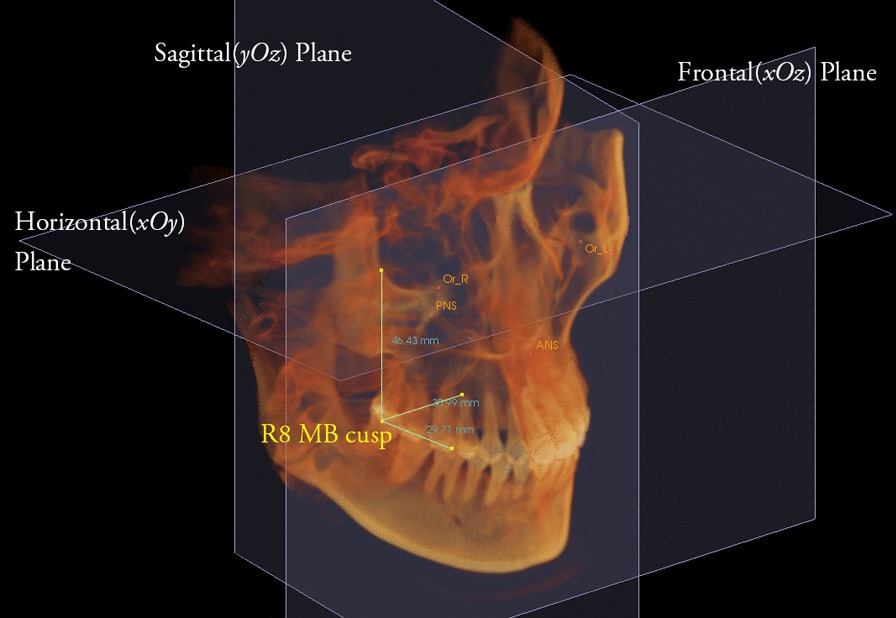
Determination of the position of right third molar. The distance from the mesiobuccal cusp to sagittal, frontal and horizontal planes were measured. R8, right third molar; MB, mesiobuccal

The distances from the mesiobuccal and distobuccal cusp of the third molar to the horizontal (xOy), frontal (xOz), and sagittal planes (yOz) were measured (Fig. 4). The changes in vertical, transverse, and sagittal positions were calculated using the distance differences before and after orthodontic treatment.

**Fig. 5:**
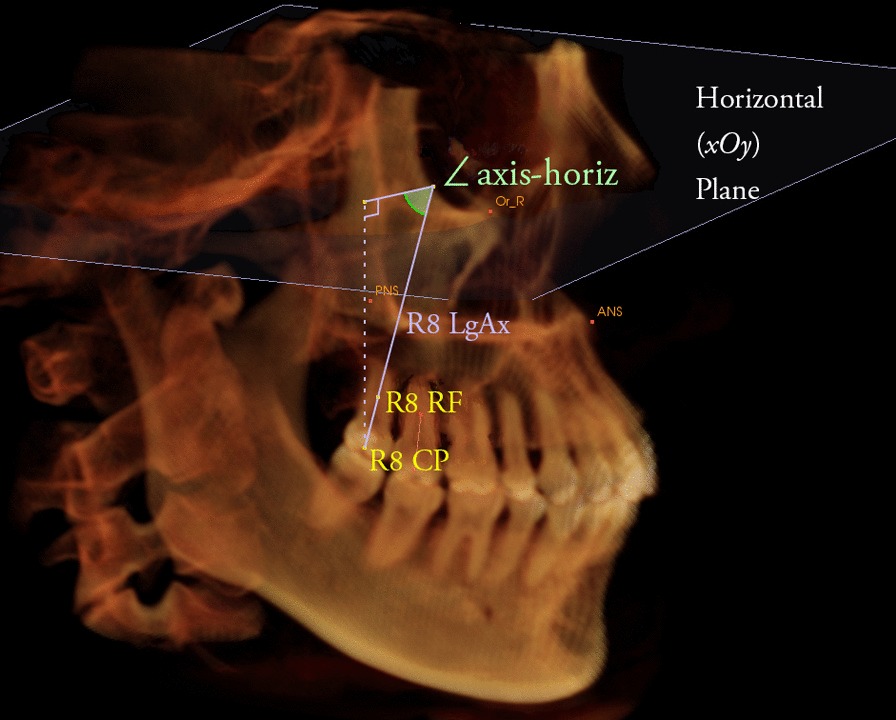
Determination of the angulation of right third molar. The angle between the long axis and the horizontal plane were measured. R8, right third molar; CP, central pit; RF, root furcation; LgAx, long axis; horiz, horizontal plane

The angles between the long axes of second and third molar (root furcation–central pit), and the angles between the long axes of third molar and the horizontal, frontal, and sagittal planes were measured (Fig. 5). Changes of the angulation of third molars in all directions before and after orthodontic treatment were calculated.

**Fig. 6:**
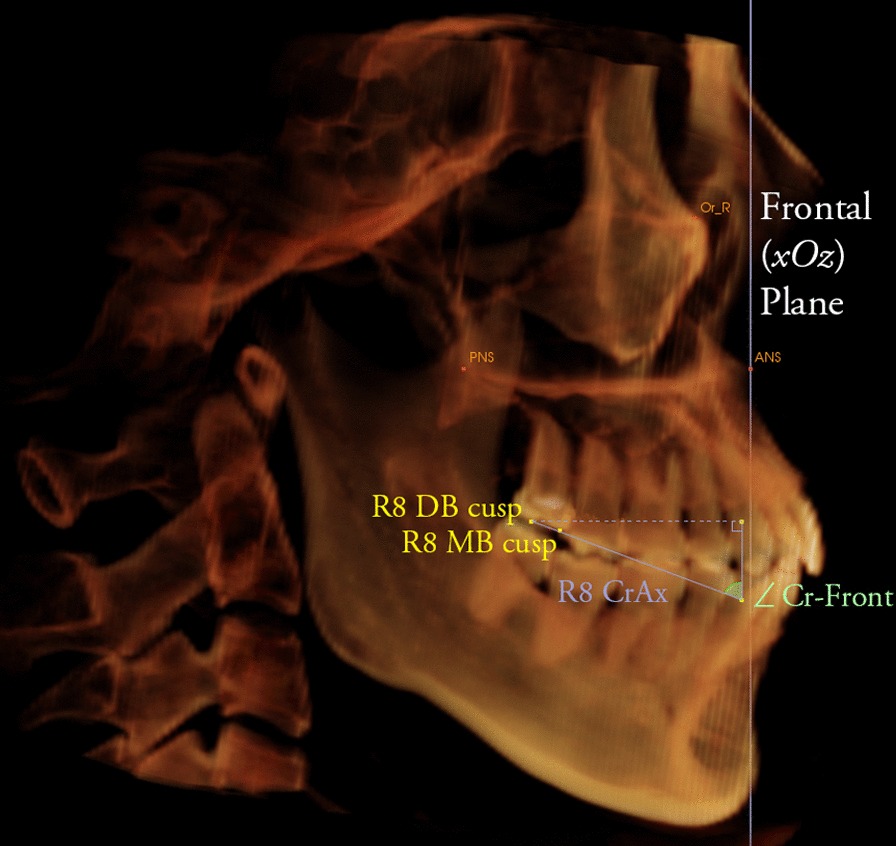
Determination of the rotation of right third molar. The angle between the mesiobuccal-distobuccal cusp (crown axis) and the frontal plane were measured. R8, right third molar; MB, mesiobuccal; DB, distobuccal; Cr, crown; Ax, axis; Front, frontal plane

The angles between the mesiobuccal–distobuccal cusp (crown axis) and the horizontal, frontal, and sagittal planes were measured (Fig. 6). Changes of the angulation before and after orthodontic treatment were measured to evaluate the rotation of third molars.

All subjects were measured independently by two operators (Y. Z. and P. F.) and both two operators were in good command of orthodontic theory and well trained in pre-experiment. No more than two patients were analyzed per day by each operator, and the whole process of evaluation was finished within 3 weeks. An experienced orthodontist (L. W.) strictly guided the study. The intraclass correlation coefficient (ICC) values ranged from 0.78 to 0.96 for all the angular variables, and from 0.96 to 0.97 for all the positional values, showing the measurement’s reliability.

Paired *t-*tests were performed to evaluate the changes between pre- and after-treatment CBCT images with SPSS software (Version 21.0; SPSS, Chicago, IL, USA). Multiple regression analysis was later processed to control heterogeneity brought by retrospective design with R software (Version 4.0.0; R, Las Vegas, USA). Statistical significance was set at 0.05.

## Results

Fourty-nine maxillary third molars from 27 patients were selected for analysis. About 22 patients had both maxillary third molars and 5 patients had unilateral maxillary third molars. Twenty-six patients received bilateral premolar extraction, and one received unilateral extraction. Table 1 shows the distribution of subjects, including demographic characteristics, orthodontic diagnoses, and treatment characteristics.

**Table 1:** Baseline characteristics of subjects

Patients (n)	27
Third molar (n)	49
Age at T1 (y)	Mean 20.78 SD 2.78
Root development at T1	
Closed apex	22 (44.90%)
Open apex	27 (55.10%)
Sex	
Male	2 (7.40%)
Female	25 (92.60%)
Treatment duration (mo)	Mean 31.47 SD 8.21
Malocclusion type	
Class I	7 (25.92%)
Class II	15 (55.56%)
Class III	5 (18.52%)
Median line (mm)	Mean 0.91 SD 0.77
Degree of crowding	
Mild	12 (44.44%)
Moderate	6 (22.22%)
Severe	9 (33.33%)
Degree of anchorage	
Maximum	21 (77.78%)
Moderate	6 (22.22%)
Tooth extracted	
First premolar	36 (73.47%)
Second premolar	13 (26.53%)
Orthodontic appliance	
Clear aligner	4 (14.81%)
Fixed appliance	23 (85.19%)

The position of maxillary third molars had significant differences after orthodontic treatment with premolar extraction (*P* < 0.001; Table 2). The third molars moved 2.87 mm downward and 1.44 mm forward respectively. On the transverse position, the mesial-buccal cusp of the third molars showed no significant difference; however, distal-buccal cusp of the third molars moved 0.69 mm outward (*P* = 0.007), which was in accordance with the result of third molars rotation (Table 2). Third molars were found to be rotated outward 5.38° (*P* = 0.001). However, the angulation of the third molars showed no difference between pre- and post- orthodontic treatment. The degree of landmarks fit of the superimposed CBCT images of reconstructed pre- and post- therapy confirmed the stability of the space coordinate system.

**Table 2:** Position and angulation of third molar compared between pre- and post- therapy

	Pretreatment		Posttreatment		Treatment effect		*P* value
	Mean	SD	Mean	SD	Mean	SD	
*Angulation (°)*							
U7 axis with U8 axis	13.28	8.97	13.53	10.08	0.25	0.40	0.854
U8 axis with horiz	62.76	15.13	63.51	14.39	0.75	10.92	0.632
U8 axis with sagit	16.85	10.40	15.21	10.32	−1.63	10.27	0.272
U8 axis with front	20.13	15.24	19.90	15.22	−0.23	9.90	0.872
*Rotation (°)*							
U8 Cr with horiz	18.35	12.48	15.91	8.02	−2.45	10.76	0.118
U8 Cr with sagit	21.20	12.14	17.21	14.67	−3.98	10.35	0.01*
U8 Cr with front	59.24	10.70	64.62	11.64	5.38	10.43	0.001†
*Position (mm)*							
U8 MB cusp to front	−40.83	3.16	−39.39	3.67	1.44	1.83	< 0.001‡
U8 MB cusp to horiz	−36.49	6.51	−39.36	6.51	−2.87	2.26	< 0.001‡
U8 MB cusp to sagit	31.09	2.49	31.16	2.17	0.07	1.34	0.702
U8 DB cusp to front	−45.02	3.15	−43.52	3.56	1.50	1.71	< 0.001‡
U8 DB cusp to horiz	−35.50	6.49	−38.60	6.44	−3.10	2.00	< 0.001‡
U8 DB cusp to sagit	29.51	2.86	30.20	2.52	0.69	1.72	0.007†

*U7* maxillary second molar, *U8* maxillary third molar, *Cr* crown, *horiz* horizontal plane; *front* frontal plane, *sagit* sagittal plane, *MB* mesial buccal, *DB* distal buccal

*p < 0.05; †*p* < 0.01; ‡*p* < 0.001

Tables 3 and 4 show the multiple regression analysis outcome. Confoundings brought by retrospective design, such as median line position, anchorage degree, orthodontic application, tooth extraction pattern, crowding degree, and malocclusion type didn’t make a significant difference in the changes of the maxillary third molars. The multi-factor analysis results are in accordance with the preceding results.

**Table 3:** Multiple regression analysis of maxillary third molar spatial position

	MB.cusp.to.sagit		MB cusp.to.front		MB.cusp.to.horiz		DB.cusp.to.sagit	
	*β*	*p*	*β*	*p*	*β*	*p*	*β*	*p*
Before-after (after)	0.07	0.701	1.44	< 0.001^‡^	−2.87	< 0.001^‡^	0.69	0.006^†^
Median line	−0.22	0.416	−0.06	0.89	0.25	0.753	−0.08	0.797
Anchorage (maximum)	0.5	0.615	1.38	0.373	0.75	0.801	0.18	0.874
Orthodontic appliance (fixed)	−0.28	0.799	0.41	0.805	4.03	0.212	−0.35	0.781
Tooth extraction (second premolar)	0.95	0.206	−0.85	0.459	−2.64	0.234	1.53	0.078
Crowding (moderate)	0.43	0.738	−2.27	0.259	2.42	0.531	−0.21	0.891
Crowding (severe)	−0.26	0.76	−0.84	0.53	1.73	0.502	−0.75	0.455
Malocclusion type (Class II)	−1.04	0.371	−1.29	0.473	3.77	0.276	−1.34	0.319
Malocclusion type (Class III)	−2.06	0.108	−0.57	0.77	4.21	0.267	−2.41	0.104

*horiz* horizontal plane, *front* frontal plane, *sagit* sagittal plane, *MB* mesial buccal, *DB* distal buccal

*p < 0.05; †*p* < 0.01; ‡*p* < 0.001

**Table 4:** Multiple regression analysis of maxillary third molar rotation

	U8.Cr.with.horiz		U8.Cr.with.front		U8.Cr.with.sagit		
	*β*	*p*	*β*	*p*	*β*		*p*
Before-after (after)	−2.45	0.115	5.38	0.001^†^	−3.98		0.008^†^
Median line	−0.47	0.653	0.76	0.549	−0.32		0.831
Anchorage (maximum)	−10.35	0.009^†^	6.08	0.197	3.24		0.554
Orthodontic appliance (fixed)	4.16	0.323	1.29	0.799	−12.7		0.034^*^
Tooth extraction (second premolar)	3.32	0.251	2.21	0.527	−2.88		0.481
Crowding (moderate)	−5.27	0.297	0.46	0.94	5.00		0.482
Crowding (severe)	1.13	0.736	−1.61	0.693	−1.77		0.708
Malocclusion type (Class II)	−5.91	0.192	1.12	0.837	4.88		0.443
Malocclusion type (Class III)	−7.47	0.133	0.46	0.938	12.68		0.071

*Cr* crown, *horiz* horizontal plane, *front* frontal plane, *sagit* sagittal plane, *MB* mesial buccal, *DB* distal buccal

*p < 0.05; †*p* < 0.01; ‡*p* < 0.001

## Discussion

In the study, a space coordinate system based on three-dimensional CBCT images taken pre- and post-treatment was established to offer more precise and comparable evaluation on the effect of premolar extraction in orthodontics on the spatial position, rotation, and angulation of maxillary third molars. Measurements used in this study were based on previous work by Lee [14], who evaluated the change of maxillary third molars after total arch distalization with a space coordinate system re-orientated by six landmarks: bilateral porions, right orbitale, ANS, nasion, and PNS.

Considering the radiation dose and financial costs, CBCT images taken for clinical orthodontic needs generally do not include cranial anatomical structures, such as nasion and sella, which add challenges to the establishment of the space coordinate system by limited time-stable lower anatomical landmarks. To address this issue, our study innovatively used only four easily recognizable landmarks: left orbitale, right orbitale, ANS, and PNS. Their stability has been confirmed in previous studies [13–15]. Superimposition according to these four landmarks was performed in this study pre- and post- treatment, confirming the stability of this space coordinate system and suggesting its reliability under low radiation dose.

The results in the current study revealed that the third molars moved downward, outward, and forward after orthodontic treatment with premolar extraction, while previous study indicated the maxillary third molars erupt downward, backward, and often outward [14], which moved in the same directions with the current study except in sagittal direction. As previous studies suggested, the consistence renders the premolar extraction a positive influence on the eruption space of maxillary third molars in orthodontic treatment [11, 16–18]. The sagittal difference can be explained by the subsequent mesial drift of third molars because of the increase of retromolar space during space closure caused by mesial drift of the molar segment [19]. However, the amount of mesial movement was smaller than the 2.2 mm mesialization of third molars previously reported [17]. The difference can be attributed to the lower potential of spontaneous tooth movement in adults [20] and relatively higher degree of anchorage.

Statistically significant improvement in the angulation of maxillary third molars was observed in previous studies [21, 22]. However, no significant difference was found in this study. This may be due to the wide use of micro-implant anchorage in the current study, including 77.78% of selected cases having received maximum anchorage, such as mini-implant and Nance arch. It has been reported that the upright of third molars might be influenced by different anchorages used in orthodontic treatment and in cases with maximum anchorage, no upright of third molars was found [23]. Besides, naturally, uprighting of maxillary third molars from initial distal inclination performs between the age of 10 and 21 to reach normal occlusal position [11]. However, the mean age of samples in the study was 20.78 years old, which may adversely affect the potential to process uprighting.

Anyhow, the revealed tendency of third molar movement after orthodontic treatment with premolar extraction does suggest reduced surgical operation difficulty and possibility of complications. Extraction should be performed at appropriate time [24]. Because oroantral perforation, the main fateful complication of maxillary third molar extraction, is closely related to the distance from the roots to sinus floor [25, 26], the downward tooth movement may lower the occurrence. Moreover, mesio-drift of maxillary third molars may help to acquire clearer view and more operation space. Thus, orthodontists could take third molars movement potential into consideration when deciding extraction plans.

The major limitation of our study was the sex distribution in the samples. Only two males were selected, probably due to disparate willingness of adult males and females to undergo orthodontic treatment. It was also a pity that heterogeneity among included samples was inevitable because of retrospective design.

Our findings could be helpful in understanding the changes of maxillary third molars after orthodontic treatment with premolar extracted. After premolar extracted and space closure, third molar movement was forward, downward, and outward, which was in favor of eruption and the alleviation of unfavorable impacted situations. This result may be clinically helpful when making a tooth extraction plan. Further well-designed prospective studies are still expected to provide more robust evidence on this topic.

Moreover, the successful practice of establishing a space coordinate system based on CBCT images using just four lower stable landmarks provides a new idea to describe the spatial location changes of certain sclerous structures. We encourage more application of the system in future studies which involve with precise superimposition and evaluation of CBCT images.

## Conclusions

This was the first study to systematically examine the spatial position changes of maxillary third molars in adult patients receiving orthodontic treatment with premolar extraction. During the process, maxillary third molars moved downward and forward accompanied by outward rotation of the crowns. Orthodontists could take tooth movement potential into consideration when deciding extraction plans. We also demonstrated the reliability and feasibility of the space coordinate system based on CBCT images.

## Data Availability

The raw data is present in the CBCT software of our university clinic. The datasets used or analysed during the current study are available from the corresponding author on reasonable request.

## Abbreviations

CBCT: Cone-bean computed tomography
T1: Before treatment
T2: After treatment
ANS: Anterior nasal spine
PNS: Posterior nasal spine
xOy: The horizontal plane
yOz: The sagittal plane
xOz: The frontal plane
U7: Maxillary second molar
U8: Maxillary third molar
Cr: Crown
horiz: Horizontal plane
front: Frontal plane
sagit: Sagittal plane
MB: Mesial buccal
DB: Distal buccal
